# Approaches to improving breast screening uptake: evidence and experience from Tower Hamlets

**DOI:** 10.1038/sj.bjc.6605393

**Published:** 2009-12-03

**Authors:** K W Eilbert, K Carroll, J Peach, S Khatoon, I Basnett, N McCulloch

**Affiliations:** 1 Public Health Directorate, NHS Waltham Forest, 7 Kirkdale House, London E11 1HD, UK; 2 McKinsey & Company, 1 Jermyn Street, London, UK; 3 Public Health Directorate, NHS Tower Hamlets, Aneurin Bevan House, 81 Commercial Road, London, UK; 4 North London Cancer Network, 5th Floor East (UCLH), 250 Euston Road, London, UK

**Keywords:** breast screening, primary care, community intervention

## Abstract

This paper reports on an innovative whole-systems approach to improving uptake of breast screening in Tower Hamlets, a deprived borough in the East End of London with a large minority ethnic population. The approach, developed by the public health team at NHS Tower Hamlets, draws on analysis of needs and existing literature about effective interventions to promote breast screening. Social marketing research led to a campaign targeted at Bangladeshi women, together with a range of initiatives to promote breast screening through primary care services and community outreach through local well-known organisations. The breast screening service itself was upgraded and a new service specification is being introduced from April 2009.

Breast screening provides an opportunity to diagnose cancer before symptoms develop, when the tumour is small and at an early stage that is likely to be associated with a good outcome. The NHS Breast Screening Programme (BSP) was introduced in the late 1980s after publication of randomised clinical trials showing that screening reduces mortality from breast cancer (Breast Screening Frequency Trial Group, 2002).

Initially, the NHS BSP was targeted at women aged 50–64 years, but since 2004 it has been extended to include women aged 50–70 years and improved by undertaking two-view mammography at all visits. The aim is to screen all women at 3-yearly intervals. Further improvements to the screening programme were announced as part of the Cancer Reform Strategy (Department of Health, 2007).

The NHS BSP is regarded as highly successful. Screening is generally considered to have contributed significantly to the overall fall in breast cancer mortality in the United Kingdom over the past 20 years (Breast Screening Frequency Trial Group, 2002). Over the past decade, the number of screen-detected cancers has doubled from 6900 per year to 14 100 per year (Health and Social Care Information Centre, 2009), and 5-year survival for screen-detected cancers is 96.4% (Health and Social Care Information Centre, 2007). However, the efficiency and effectiveness of breast screening varies across the country, with particular problems in London:

- Overall coverage (the proportion of eligible women who have been screened at least once in the previous 3 years) is 77% nationally, but only 65% in London (Health and Social Care Information Centre, 2009).
- Uptake (proportion of women invited for whom a screening result is recorded within 6 months) is 73% nationally, but only 61% in London (Health and Social Care Information Centre, 2009).
- Round length (the proportion of eligible women whose first offered appointment is within 36 months of their previous screening) is 84% nationally, but only 60% in London (Health and Social Care Information Centre, 2007).

We describe a multifaceted, whole-systems approach developed by NHS Tower Hamlets Public Health to improve coverage, uptake and round length of breast screening and thereby to decrease breast cancer mortality in Tower Hamlets. This paper draws on existing literature about effective approaches, presents new analyses undertaken to understand the specific problems in Tower Hamlets, describes the interventions that have already been undertaken and further interventions to be carried out in 2009, and presents data on screening uptake.

## Factors that may contribute to low coverage

**Figure 1:**
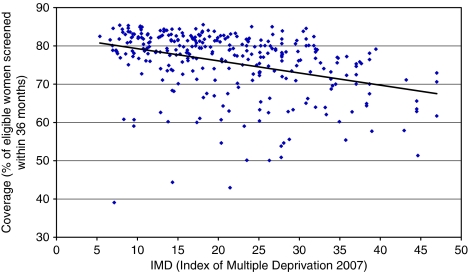
The relationship between deprivation and breast screening coverage at a PCT level (*r*^2^=0.13). *Source*: Office for National Statistics (2003) and Health and Social Care Information Centre (2007).

Deprivation is a significant factor in explaining low breast screening coverage, but only contributes around 13% of the variation in coverage at Primary Care Trust (PCT) level. Some PCTs have high coverage despite severe deprivation. Figure 1 shows the relationship between deprivation and breast screening coverage at a PCT level. Within PCTs, coverage at a general practitioner (GP) practice level is not linked to deprivation of their catchment area (based on unpublished Mori poll data for Tower Hamlets, Newham, City and Hackney, Waltham Forest, Islington, Camden PCTs).

**Figure 2:**
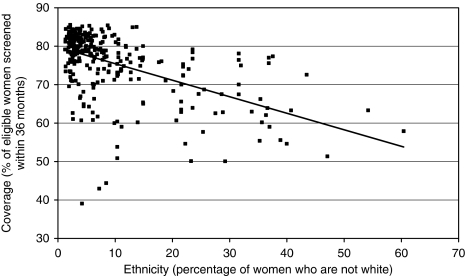
The relationship between ethnicity and breast screening coverage at a PCT level (*r*^2^=0.28). *Source*: Office for National Statistics (2003) and Health and Social Care Information Centre (2007).

Ethnicity accounts for around 28% of the variation between PCTs as shown in Figure 2. There are significant differences in uptake between minority ethnic groups within the area covered by the Central and East London Breast Screening Service (CELBSS). Unpublished Dr Foster Intelligence work for CELBSS indicated that uptake is 60% in the white population and a similar figure is seen for the Indian population, whereas uptake is much lower in Pakistani (40%) and Bangladeshi (37%) populations. This may be caused by cultural, linguistic or other factors. In Tower Hamlets, 55% of the population of screening age (50–70 years) are white, 1% is Pakistani and 27% are Bangladeshi. To improve coverage levels, it is very important to work with all of these communities.

**Figure 3:**
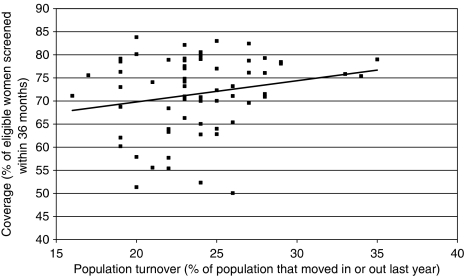
The relationship between population turnover and breast screening coverage at a PCT level (*r*^2^=0.04). *Source*: Bailey and Livingston (2007). Office for National Statistics (2003) and Health and Social Care Information Centre (2007).

Population turnover in Tower Hamlets is marginally above that for London and 4% higher than that for England (Office for National Statistics, 2003). High population turnover is commonly thought to contribute to low screening coverage, but this is not supported by analysis of coverage and total population turnover at a PCT level (Bailey and Livingston, 2007). However, the measure of total population turnover may not accurately reflect changes in the eligible screening population, as most turnovers are in people under 45 years. Population turnover accounts for about 4% of the variation in breast screening coverage as shown in Figure 3.

The importance of deprivation, ethnicity and population turnover, as well as other factors such as literacy levels and social integration, is reviewed elsewhere (Sutton *et al*, 1994; Champion and Scott, 1997; Box, 1998; Raja-Jones, 1999; Oelke, 2002; Chui, 2003).

## Materials and methods

The whole-systems approach taken by Tower Hamlets includes interventions aimed at targeted outreach work through respected community organisations to the eligible population (women aged 50–70 years), improving the breast screening service and strengthening commissioning.

### Targeted outreach through respected community organisations

Previous studies undertaken in groups of women with low rates of screening, including Asian/Indian women, have shown the positive effects of knowledge building (Sadler *et al*, 2001), telephone counselling (Rimer *et al*, 2002), person-to-person conversations (Legler *et al*, 2002), professional encouragement (Legler *et al*, 2002) and peer support (unpublished work by Straight Talk). Among Bangladeshi women, the belief is that cancer is a death sentence and language barriers may be particular problems (identified in unpublished work by the social marketing company Forster for Change for Tower Hamlets PCT).

Building on this, Tower Hamlets PCT worked with Forster for Change to develop a campaign targeted at both Bangladeshi and white women. The team worked with an influential community group, Social Action for Health, to support Bangladeshi women to attend screening appointments. This involved calling women who did not attend their appointments, rebooking their appointments for them and, in some cases, providing transport for groups of women to attend. Of 219 women involved in this pilot, 151 (69%) went on to attend screening. Meanwhile, a local woman was appointed to lead a campaign for white women, giving positive messages about attending breast screening as part of taking care of their health. A Bosom Buddy pilot is now starting to encourage women who have been screened to recruit a friend or family member who has not attended, and help them through the appointment.

Measures have also been developed to help women who have low literacy or are unfamiliar with English. Talking invitations were developed for women who cannot read or use a spoken-only dialect such as Sylheti, and these will be tested from June 2009. A pilot was implemented in two GP practices to call women before they receive their invitations and encourage them to attend their screening appointments, helping those who cannot read to make an informed choice about attendance. Support and translation is also provided through the Tower Hamlets PCT health advocates service at the static breast screening unit.

### Primary care services

Tower Hamlets PCT has instituted a range of interventions, based on published evidence where available, to promote breast screening through primary care services. A Local Enhanced Scheme was launched in 2007/2008 to incentivise GPs to increase participation in screening. Payment was based on the number of additional eligible women screened within the practice.

Training was provided to all 20 GP practices that formed the 2007/2008 screening round. Public health teams visited each practice three times, accompanied by a representative of the local breast screening service on the first visit. The benefits of screening were explained to staff, as recommended by Gorin *et al* (2007), and the roles of individuals within the practice team in increasing screening uptake were explored, as recommended by Atri *et al* (1997).

A Bangladeshi GP screening lead was appointed for the PCT to raise the profile of all three cancer screening services, to front the campaign to advise Bangladeshi women to attend breast screening and to work with Public Health to encourage best practice in primary care for cancer screening in GP practices. After recommendations of a Best Practice Cancer Screening Guide for Primary Care developed by the Public Health Directorate in NHS Tower Hamlets, each general practice also nominated a cancer screening lead (a practice manager or a GP).

To encourage uptake, GP practices set up alerts for eligible women to provide opportunistic reminders when they attended the clinic for other reasons. They also endorsed invitation letters, as recommended by Turner *et al* (1994), established ‘well-women’ pilots to provide ‘prescriptions’ for breast screening and sent reminders through text messages. While the service only had mobile phone numbers for 10% of women, 70% of those who received messages attended their screening appointments (unpublished evaluation of test messaging services by iPLATO in 2008).

### Breast screening service

As part of the whole-systems approach to improving breast screening in Tower Hamlets, it was recognised that attention should be given to improving the efficiency and effectiveness of the screening service itself. An investigation in 2004 by Sue Hudson had shown that on average 830 calls per day are made to the breast screening services in London, and of these, 37% were engaged or unanswered. This can lead to a significant number of appointments not being attended as on average 23% of appointments are cancelled or changed by phone.

McKinsey and Company was engaged to work with the CELBSS to address this and other efficiency issues. As a result, CELBSS established a dedicated call centre; the missed call rate dropped by 38% within 2 weeks. This meant, for example, that women who called were more likely to be able to change their appointments.

Interactions with radiographers during a screening appointment can influence a woman's decision to return. On that basis, customer service training was provided for all staff, including radiographers and daily team meetings were set up with the superintendent radiographer. A service charter, developed by CELBSS staff, will be put on display at each screening site, setting out what women can expect during their appointment in terms of waiting time, access to interpreters and friendly and respectful staff.

The service now provides second timed appointments for non-attenders (from April 2009), as these have been shown to increase attendance rates in comparison with an open invitation to call the service (Stead *et al*, 1998). Capacity was increased to ensure that round length targets are achieved (see next section).

### Stronger commissioning

The CELBSS is commissioned jointly by six PCTs working through a lead PCT in Tower Hamlets. A new service specification for the breast screening service has been introduced from April 2009. This focused on quality measures of patient experience and included a tariff-based payment scheme that incentivises the provider and allows for increased capacity.

## Results

Tower Hamlets PCT has historically had low participation rates in breast screening. Detailed analysis showed that there were multiple reasons for this. A whole-systems approach, based on evidence of effective interventions, has been implemented. Preliminary findings indicate significant improvements both in processes and in uptake. In 2005, uptake was 44.5%. Three years later, this had risen to 58.1% and in 2008/2009, the figure was 63.4%. These increases have not been analysed for statistical significance.

## Discussion

A multi-pronged approach through a strong partnership between community organisations, GP practices and public health with targeted promotion, along with attention to the quality of the service provided, seems to be effective in increasing breast screening rates.

There are several potential areas for improvement. Text messaging can only be as good as the availability of mobile numbers. In the participating GP practices, only 10% of mobile numbers were available. As text messaging is an effective way of managing overall appointments, GP practices could work to collect mobile numbers for the next screening round. Community outreach worked well with the Bangladeshi community. There is still need to develop an appropriate outreach mechanism for the white community. A Bosom Buddy scheme is being piloted to test a mechanism to reach white women. There is still work to do to involve GPs more in the screening process, including understanding the variation between GP practice rates (range from 79.3% to 35.9%).

The measure of success for each separate activity was women's attendance for their breast screening appointments. This was measured by providing a card to each person participating in an activity that she then turned in if she attended her appointment. As there were a number of interventions run at the same time, it was not possible to say that any single one caused a woman to attend.

While some interventions were based on evidence from the literature, where there was no evidence, pilots were used to test interventions. As success was measured solely by attendance without controlling for possible intervening factors, it is not possible to generalise findings to other settings.

### Conclusion

Working in a community setting to influence behaviour change poses challenges to research that may call for practical approaches where resources may be limited and it would not be possible to control for all factors that influence women's decision making. Combining a systematic approach based on existing evidence with innovative interventions led to useful lessons in Tower Hamlets that can be applied to improve uptake of this important public health screening programme and thus the health of women of Tower Hamlets.

## Conflict of interest

The authors declare no conflict of interest.
